# Postpartum management of bipolar disorder: pharmacological and psychosocial strategies for relapse prevention and recovery

**DOI:** 10.1192/j.eurpsy.2026.10283

**Published:** 2026-07-31

**Authors:** T. Kurimay

**Affiliations:** Psychiatry and Psychatric Rehabilitation, Teaching Dep. of Semmelweis University, North-buda Saint John Central Hospital Family Centred Mental Health Centre, Budapest, Hungary

## Abstract

**Abstract:**

The postpartum period represents one of the highest-risk phases for relapse in women with bipolar disorder, with a markedly increased vulnerability to depressive episodes, mania, mixed states, and postpartum psychosis. Rapid biological changes after childbirth, combined with sleep deprivation, stress, and psychosocial demands, can destabilize even previously well-controlled patients. Effective postpartum management therefore requires proactive, individualized planning that integrates pharmacological relapse prevention with psychosocial interventions and family-centred care.

This presentation provides practical, evidence-based guidance for everyday clinical practice in postpartum bipolar disorder. Pharmacotherapy remains a cornerstone of relapse prevention. Lithium is among the most effective options, particularly for preventing severe postpartum mood episodes; however, careful monitoring is essential due to postpartum physiological changes affecting fluid balance and renal clearance. Other mood-stabilizing strategies may include lamotrigine and second-generation antipsychotics, selected according to prior response, symptom profile, and tolerability. Treatment decisions must also address breastfeeding-related concerns. Shared decision-making is crucial, balancing the benefits of breastfeeding with maternal stability, infant safety, and the risk associated with sleep loss. In acute episodes with psychotic symptoms or severe agitation, rapid stabilization and safety planning—including consideration of hospitalization and, when indicated, electroconvulsive therapy—may be lifesaving.

Alongside medication, psychosocial interventions are essential and should not be considered optional. Psychoeducation focused on relapse signatures, early warning signs, and adherence support improves insight and timely help-seeking. Sleep protection is a key preventive strategy, requiring structured plans for night-time support and realistic expectations around infant care. Interpersonal and social rhythm approaches may help restore daily routines and reduce mood instability, while cognitive-behavioural techniques can support coping with anxiety, guilt, and intrusive thoughts. Partner and family involvement is often critical for monitoring symptoms, reducing burden, and strengthening protective factors. Special attention should be given to the parent–infant relationship, as mood episodes may impair bonding and parenting confidence; supportive interventions can promote recovery and healthier developmental outcomes.

Overall, integrated postpartum care combining pharmacotherapy with psychosocial and family-based strategies can reduce relapse risk, improve maternal functioning, and support safe, meaningful early motherhood for women living with bipolar disorder.

**Disclosure of Interest:**

None Declared

